# Feasibility and Preliminary Efficacy of a Guided Self‐Help Digital Intervention for Adults With Food Insecurity, Recurrent Binge Eating, and Type 2 Diabetes Mellitus: A Pilot Trial

**DOI:** 10.1002/eat.70087

**Published:** 2026-03-23

**Authors:** Andrea K. Graham, Rebecca L. Flynn, Isabel R. Rooper, Chidiebere Azubuike, Lindsay D. Lipman, Adrian Ortega, Leah M. Parsons, Graham C. Miller, Damien Lekkas, Jennifer E. Wildes

**Affiliations:** ^1^ Center for Behavioral Intervention Technologies, Northwestern University Feinberg School of Medicine Chicago Illinois USA; ^2^ Department of Medical Social Sciences Northwestern University Feinberg School of Medicine Chicago Illinois USA; ^3^ Department of Preventive Medicine Northwestern University Feinberg School of Medicine Chicago Illinois USA; ^4^ Department of Psychiatry & Behavioral Neuroscience The University of Chicago Chicago Illinois USA

**Keywords:** binge eating, diabetes, digital intervention, eating disorders, food insecurity, human‐centered design, pilot trial

## Abstract

**Objective:**

Food insecurity is increasingly linked to binge eating and weight‐related health issues like type 2 diabetes mellitus, but no eating disorder interventions have been tested among individuals with food insecurity. We conducted a single‐arm pilot test of FoodSteps‐FI, a guided self‐help digital intervention for binge eating adapted for food insecurity, among adults with food insecurity, recurrent binge eating, and type 2 diabetes mellitus. Our aims were to test the feasibility and preliminary efficacy of FoodSteps‐FI delivery.

**Method:**

Participants were 31 non‐pregnant, English‐speaking adults (≥ 18 years old) with self‐reported type 2 diabetes mellitus, recurrent binge eating (≥ 12 episodes in the past 3 months), and past 30‐day food insecurity who were interested in intervention and owned a smartphone with internet access. All participants received FoodSteps‐FI with messaging‐based coaching (~2×/weekly) for 16 weeks, and a weekly stipend. Participants completed assessments at mid‐intervention (8 weeks) and post‐intervention (16 weeks). Intent‐to‐treat analyses assessed feasibility and changes in clinical outcomes from baseline to post‐intervention.

**Results:**

The trial demonstrated feasible recruitment, high study retention, and high intervention completion, adherence, and ratings of usability. Participants achieved improvements in binge eating, weight, past 30‐day food security status, eating concerns, shape concerns, weight concerns, eating disorder impairment, depressive symptoms, shame, and guilt. Nearly all participants rated the stipend as helpful for making behavior changes, but substantially fewer said it was sufficient.

**Discussion:**

This pilot trial demonstrated the early promise of an adapted digital binge‐eating intervention for this population, and the need for future research on its effectiveness and implementation.

## Summary

- There is a growing understanding of the association between food insecurity and binge eating, and a striking lack of intervention.
- We pilot tested a digital intervention for binge eating, adapted for food insecurity, among adults with food insecurity, recurrent binge eating, and type 2 diabetes mellitus.
- Pilot results showed improvements in binge eating, weight, food security status, eating disorder psychopathology, depressive symptoms, shame, and guilt.
- Future research is needed to evaluate this intervention in a fully powered trial with a larger sample compared to a controlled condition.

## 1 Background

Food insecurity, defined as inadequate or inconsistent access to the food required for a healthy life (Barrett 2010; Coleman‐Jensen et al. 2022), is increasingly linked to excess weight/obesogenic environments (Myers et al. 2020; Pryor and Dietz 2022) as well as eating disorder (ED) psychopathology including binge eating (Abene et al. 2023; Hazzard et al. 2020) and weight‐related health issues like type 2 diabetes mellitus (T2DM; Levi et al. 2023). Intervention to address binge eating and weight‐related behaviors is needed for this underserved population given the unique presentation of binge eating in this group (e.g., onset of binge eating with financial cycles, higher stress‐related eating, limited resources and access to care). Yet until the present study, no such intervention has been developed and tested. To address this gap, we adapted and pilot tested FoodSteps‐FI, a guided self‐help cognitive‐behavioral digital intervention for binge eating and weight‐related behaviors, among adults with food insecurity, recurrent binge eating, and T2DM. A digital intervention could overcome structural barriers to accessing ED care, such as that most evidence‐based treatments are offered in specialty care centers by trained clinicians in larger cities, which can disadvantage individuals from lower socioeconomic strata (Radunz et al. 2021), who experience elevated rates of food insecurity (Odoms‐Young et al. 2024). Further, a digital intervention can deliver in‐the‐moment support that could disrupt how binge eating presents in this population.

The original intervention, FoodSteps, created from the start as a digital intervention, was previously designed with and tested among adults with recurrent binge eating and obesity (Graham et al. 2021), some of whom had food insecurity and/or T2DM, though these characteristics were not inclusion criteria. In this pilot trial, we specifically enrolled individuals with T2DM. This was a clinically important extension given the link between food insecurity and T2DM, as well as the prevalence of binge eating (up to 20% of people with T2DM) and the negative impact of binge eating on physical health, mental health, and quality of life among people with T2DM (Cerrelli et al. 2005; Harris et al. 2021).

Because the original intervention did not directly address food insecurity, we anticipated adaptations would be needed. We interviewed potential end‐users of FoodSteps to learn facilitators and barriers that individuals with food insecurity believed would impact their engagement with a digital intervention (Venkatesh et al. 2021). Participants specified features they perceived would improve engagement and efficacy, including providing education on the importance of a healthy lifestyle and addressing binge eating, teaching ways to make healthy changes on a budget, personalizing goals to themselves and their finances, improving body image and self‐efficacy, having support from a coach, and offering financial resources to support implementing healthy behavior changes. Although most of those features were already part of FoodSteps' design (e.g., education on the importance of healthy eating, personalized goal setting, body image as a treatment target, coach support), responses indicated adaptations were needed. Thus, we adapted FoodSteps by integrating education specific to people experiencing food insecurity and providing a weekly stipend to improve capability to engage in the intervention. These changes aligned with the COM‐B system that posits behavior change happens when opportunity, motivation, and capability interact (Michie et al. 2011).

This study presents results from a pilot trial that tested FoodSteps‐FI among people with food insecurity, recurrent binge eating, and T2DM. Our aims were to test the feasibility (of recruitment, study retention, intervention completion and adherence, and intervention usability) and preliminary efficacy (change in binge eating [primary] and other clinical metrics) of delivering FoodSteps‐FI in this population. This was the first time an ED intervention for binge eating was tested among people with food insecurity, a group in high need of support.

## 2 Methods

The trial was pre‐registered on ClinicalTrials.gov (NCT06348251) and approved by the Northwestern University Institutional Review Board.

### 2.1 Participants

We aimed to enroll 30 participants, and acknowledge that, as a pilot, this trial was not powered to provide a stable effect size estimate and instead aimed to provide data on feasibility. Eligible participants were non‐pregnant, English‐speaking adults (≥ 18 years old) with self‐reported T2DM, recurrent binge eating (≥ 12 episodes in the past 3 months) and current food insecurity (score ≥ 2 on the six‐item 30‐day Short Form Food Security Survey; United States Department of Agriculture 2012). Among those potentially eligible at screening, we subsequently confirmed recurrent binge eating via the Eating Disorder Examination (EDE) interview (Fairburn et al. 2008). Participants could endorse objective or subjective binge eating, consistent with ICD‐11 and prior research on the association between binge eating, across episode types, in people with food insecurity (e.g., Abene et al. 2023; Becker et al. 2017; Hazzard et al. 2020). Eligible participants also needed to endorse at screening marked distress about their binge eating, desire to lose weight and reduce binge eating, and willingness to use a mobile app. Lastly, they needed to endorse access to a scale for self‐weighing, a smartphone with internet access and capacity to receive calls and texts, and a valid email address to complete study procedures. Endorsement of compensatory behaviors on the EDE was not exclusionary.

Individuals were excluded if they self‐reported a diagnosis for which the study or intervention would not be clinically indicated (e.g., psychosis, mania, cognitive impairment). To avoid confounding effects with other treatments, individuals also were excluded if they were working with a clinical professional to receive services for management of weight or binge eating, or if they started or recently changed (within the past month) dosage of a medication for weight loss, binge eating, or T2DM.

### 2.2 Procedures

Participants were recruited from February 2024 to July 2024. In‐person recruitment was conducted in Chicago, IL at our community partner sites (e.g., food pantries) and at community events (e.g., health/wellness fairs). Print advertisements were posted in public settings (e.g., health clinics, public transit stops, libraries). Participants were recruited online via social media, Craigslist, and ResearchMatch. Special attention was devoted to recruiting a racially and ethnically diverse sample to enhance generalizability. We created a study‐specific website and video for up‐front information‐sharing about the intervention and trial. We conducted design sessions to assess target participants' preferences on our recruitment materials' aesthetics (e.g., features, imagery), accessibility (e.g., language comprehension), credibility and effectiveness, and placement (e.g., multimedia location). Recruitment materials comprised images of individuals with a range of sociodemographic backgrounds (e.g., age, race, ethnicity), and different ads varied terms to describe binge eating, because end‐users had indicated they refer to their own binge eating with a variety of terms. During the trial, we applied data‐driven tracking to determine the success of recruitment pathways (e.g., via QR code scans) and when to iterate. We also applied “recruitment quotas” for different sex/racial/ethnic groups based on our planned enrollment targets, and paused or discontinued recruitment of certain groups when that group's quota was met.

Interested individuals completed online consent to screening and a brief online survey via REDCap to assess for initial eligibility. Potentially eligible participants were invited to complete a baseline assessment to confirm eligibility (i.e., written informed consent followed by an interview and REDCap surveys).

Eligible individuals were invited to enroll in the trial and use FoodSteps‐FI with coaching (see below). All participants were offered FoodSteps‐FI (i.e., no inactive placebo). At mid‐intervention (8 weeks after onboarding to FoodSteps‐FI), participants were asked to complete online surveys (i.e., the same as at baseline with the addition of questions assessing other treatment/intervention utilization and the System Usability Scale; approximately 20 min per administration). Following the intervention (“post‐intervention”; 16 weeks after onboarding), participants were invited to complete an interview and online surveys (i.e., the same as at mid‐intervention, with the addition of the Stipend Questionnaire). Research assessors were distinct from intervention coaches.

All procedures were conducted remotely. Participants received up to $90 compensation via an electronically‐delivered cash gift‐card after completing each assessment ($20 for baseline, $25 for mid‐intervention, $30 for post‐intervention, $15 bonus for completing all). As detailed below, participants also received a stipend as part of intervention delivery ($10 per week for the 16‐week intervention via an electronically‐delivered cash gift‐card), administered regardless of whether they completed study assessments. Thus, in total, participants could receive $250.

### 2.3 FoodSteps‐FI

FoodSteps, and the adapted FoodSteps‐FI version, is a 16‐week digital cognitive‐behavioral intervention for managing binge eating and weight‐related behaviors. FoodSteps' theoretical model integrates mechanisms of evidence‐based behavioral and psychological treatments to intervene on five evidence‐based treatment targets: improving healthy eating behaviors, increasing physical activity, reducing overvaluation of weight and shape, decreasing unhealthy weight control practices, and reducing negative affect (Graham et al. 2021). Weekly activities were designed to be brief (~5 min). Each week, users completed a brief self‐assessment and reviewed content on a topic related to the five treatment targets (e.g., creating an eating plan, eating in moderation, increasing social support for health behaviors, understanding the relationship between food and mood, coping with weight bias, maintaining progress, and preventing relapse). Then, users selected one of the five treatment targets to work on, set a personalized goal relevant to that target, and created a plan to practice their goal over the week ahead. During the week, they tracked their progress practicing their goal. Automated text messages supported skill practice and provided reminders to facilitate adherence. Users also could view in the app daily tips related to the treatment target they selected. These strategies to promote behavior change were derived from the behavior change technique taxonomy (Michie et al. 2013). FoodSteps‐FI did not target diabetes management beyond positive health behaviors and behavioral weight management.

Additionally, to support adherence and motivation, each user was assigned a coach (a bachelor's level non‐specialist) who was trained and supervised weekly by a clinical psychologist. Coaching followed a low‐intensity model of supportive accountability (Mohr et al. 2011) aimed at promoting engagement by encouraging users to use the intervention and practice skills in their daily lives. Coaches also provided technical support as needed. Coaches primarily used text messages to communicate with users through a secure dashboard. Coaching began with a 30‐min phone call to establish goals and build rapport, ensure the user can access the intervention, and set expectations for the coaching relationship. Thereafter, users received approximately one to two text messages per week from their coach to reinforce app use, offer encouragement, and check in on progress or challenges. Coaches responded to participant‐initiated messages within 1 business day, with no limit on the total number of messages they could receive. An optional 10‐min phone call mid‐way through the intervention (Week 8) was offered to users to discuss their experiences utilizing the program and address any concerns.

Based on feedback from individuals with food insecurity in our previous design work (Venkatesh et al. 2021), participants in FoodSteps‐FI also received a $10 weekly stipend ($160 total) to support implementing healthy behavior changes during the intervention. Participants received the stipend regardless of whether they engaged with the digital intervention, but were informed that it would be discontinued if they withdrew from the study. We determined the stipend amount in collaboration with our university's accounting office to be meaningful yet below the threshold at which participants would need to submit a W‐9 federal payment form and report these earnings. (In the United States, payments above a threshold, currently $600/year, require reporting for tax purposes.) We wanted to avoid the added burden of a W‐9 because of the sensitive information it requires (a social security number), which could trigger mistrust and its potential to influence participants' eligibility for governmental benefits.

Prior to onboarding, participants received a worksheet with ideas for how the stipend could be spent to support eating/lifestyle changes; at onboarding, coaches asked participants to generate ideas for how they might use their stipend. Subsequently, coaches did not follow up on stipend usage except during the midpoint call to see how this component was helping them meet their weekly goals.

### 2.4 Measures

Feasibility of study procedures was based on study recruitment rates and retention rates. Intervention completion was measured as the proportion of FoodSteps‐FI sessions completed out of 16 sessions. Intervention adherence was measured via weekly completion of four prescribed behavior change techniques (Michie et al. 2013): self‐reporting weight and number of binge episodes over the past week (self‐monitoring), committing to ≥ 1 goal for the week ahead (goal‐setting), and establishing a plan for ≥ 1 “high‐risk” situation that could impede their goal (action planning and problem‐solving). Intervention usability was assessed via the System Usability Scale (Bangor et al. 2008; Brooke 1996), a 10‐item questionnaire with scores ranging from 0 to 100; scores > 70 are considered passable usability, with better products scoring in the high 70s to upper 80s (Bangor et al. 2008).

Demographics (e.g., gender, Hispanic ethnicity, race, education, household income) were assessed using an investigator‐designed questionnaire. We also assessed for comorbid mental health diagnoses and for other treatments/interventions utilized over the study period (professional clinical services, medication, apps, books/workbooks). Episodes of binge eating and compensatory behaviors were assessed using the EDE, administered by a trained assessor. Outcome analyses for binge eating (regardless of episode size) were conducted using EDE‐derived episodes of binge eating over the past 28 days. For individuals who did not complete the EDE at post‐intervention, past 28‐day binge eating was obtained from the EDE Questionnaire (EDE‐Q; Fairburn and Beglin 2008).

Weight was assessed via self‐report. ED psychopathology was assessed via the EDE‐Q subscales, each scored on a 0–6 scale with higher scores indicating greater psychopathology. ED‐related clinical impairment was assessed via the Clinical Impairment Assessment (Bohn et al. 2008; Bohn and Fairburn 2008), with higher total scores indicating greater impairment. Depressive symptoms were assessed via the Center for Epidemiologic Studies Depression Scale Revised (Radloff 1977), with higher scores indicating worse depressive symptoms. Perceived stress was assessed via the Perceived Stress Scale (Cohen et al. 1983), with higher scores indicating higher perceived stress. The Personal Feelings Questionnaire‐2 (Harder and Zalma 1990) was used to assess feelings of shame and guilt. Food insecurity was assessed via the 6‐item 30‐day Short Form Food Security Survey (United States Department of Agriculture 2012). Scores of 0–1 indicate food security, 2–4 indicate low food security, and 5–6 indicate very low food security. Cronbach's alpha for relevant measures at each study timepoint is in Table S1.

At post‐intervention, participants completed a Stipend Questionnaire, a 12‐item questionnaire created by our team for this study (previously published in Graham et al. 2024). Questions assessed how the stipend was used (e.g., “Food for me,” “Food for others,” “Cooking supplies”), its helpfulness and sufficiency, and disbursement preferences, with one open‐ended question assessing for anything else they wanted to share about the stipend.

### 2.5 Statistical Analyses

Analyses were conducted using R (Posit team 2024; R Core Team 2024; Thériault 2023). One participant did not complete the post‐intervention assessment, thus their last observation carried forward (i.e., from mid‐intervention) was used to impute post‐intervention assessment values (except for the Stipend Questionnaire, which was only administered post‐intervention). Feasibility metrics were calculated using means and standard deviations. Each clinical outcome was assessed for significant change across the cohort accounting for each timepoint (baseline, mid‐intervention, post‐intervention). The *lmerTest* R package (v3.1‐3) was used to conduct linear mixed effects models with time (baseline and post‐intervention) treated as a fixed effect and participant intercept included as a random effect to control for differences across participant baselines. Post hoc multiple hypothesis correction was performed to control for the false discovery rate via Benjamini‐Hochberg. Adjusted *p* values < 0.05 were considered statistically significant. Effect size and 95% confidence intervals (CIs) for each model were quantified through ANOVA in the *effectsize* R package (v1.0.0) to derive eta‐squared.

## 3 Results

### 3.1 Sample Characteristics

Thirty‐one participants enrolled, 74% (*n* = 23) of whom self‐identified as female. Mean age was 47 years old (SD = 11.72; range = 25–75). Regarding educational attainment, 36% (*n* = 11) had at least a bachelor's degree. Regarding race and ethnicity, 16% (*n* = 5) self‐identified as Hispanic, 29% (*n* = 9) as non‐Hispanic Black, 42% (*n* = 13) as non‐Hispanic White, and 13% (*n* = 4) as non‐Hispanic and another race. At baseline, 39% (*n* = 12) had low food security and 61% (*n* = 19) had very low food security. Past 28‐day binge episode average was 12.68 (range = 2–30) and the past 3 months average was 41.48 (range = 14–109). Mean household income was $33,416.58 (SD = $23,082.12; range = $0–$99,000). Sociodemographic characteristics are in Table 1.

**TABLE 1:** Sociodemographic characteristics of study participants at baseline (*N* = 31).

Age (years), mean (SD)	47.45 (11.72)
Gender, *n* (%)	
Female	23 (74%)
Male	7 (23%)
Nonbinary	1 (3%)
Hispanic ethnicity, *n* (%)	5 (16%)
Race, *n* (%)	
Asian	1 (3%)
Black	9 (29%)
Native American or Alaska Native	3 (10%)
Pacific Islander	1 (3%)
White	17 (55%)
Education, *n* (%)	
High school diploma or less	4 (13%)
Some college	10 (32%)
2‐year degree	6 (19%)
4‐year degree or higher	11 (36%)
Household income in dollars, mean (SD)	$33,416.58 ($23,082.12)
Baseline food security, *n* (%)	
Low food security (score 2–4)	12 (39%)
Very low food security (score 5–6)	19 (61%)
Binge eating episodes (past 28 days), mean (SD)	12.68 (7.27)
Baseline weight (pounds), mean (SD)	244.97 (57.76)
Baseline BMI (kg/m^2^), mean (SD)	49.94 (16.02)
Type 2 diabetes mellitus	31 (100%)

### 3.2 Feasibility

**FIGURE 1:**
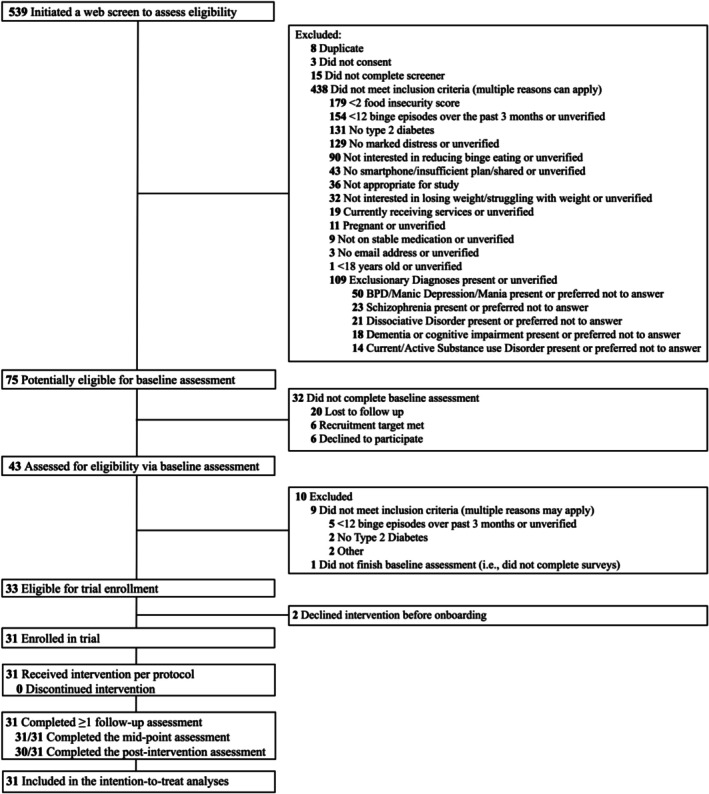
CONSORT diagram.

Figure 1 shows the CONSORT diagram. Feasibility of recruitment was achieved; we exceeded our target by enrolling 31 participants at an average rate of six participants per month. Regarding study retention, zero participants withdrew from the trial, all completed the mid‐intervention assessment, and 97% (*n* = 30) completed the post‐intervention assessment. Among those who completed the post‐intervention assessment, 87% (*n* = 27) completed both the EDE and surveys, and 10% (*n* = 3) only completed surveys.

Participants completed an average of 15.29 of 16 sessions (SD = 2.08; range = 7–16) and had high rates of adherence to intervention components. On average, participants completed self‐monitoring on 15.29 of 16 weeks (SD = 2.08), set a goal on 15.19 of 16 weeks (SD = 2.23), and engaged in planning and problem‐solving on 14.65 of 16 weeks (SD = 2.44). About half (52%; *n* = 16) completed the optional mid‐point coaching call.

Average usability score at mid‐intervention was 84.35 (SD = 16.43; median = 92.50; range = 50–100) and at post‐intervention was 82.58 (SD = 18.38; median = 88.75; range = 45–100), indicating above‐acceptable intervention usability (> 70) (Bangor et al. 2008).

### 3.3 Preliminary Efficacy

**FIGURE 2:**
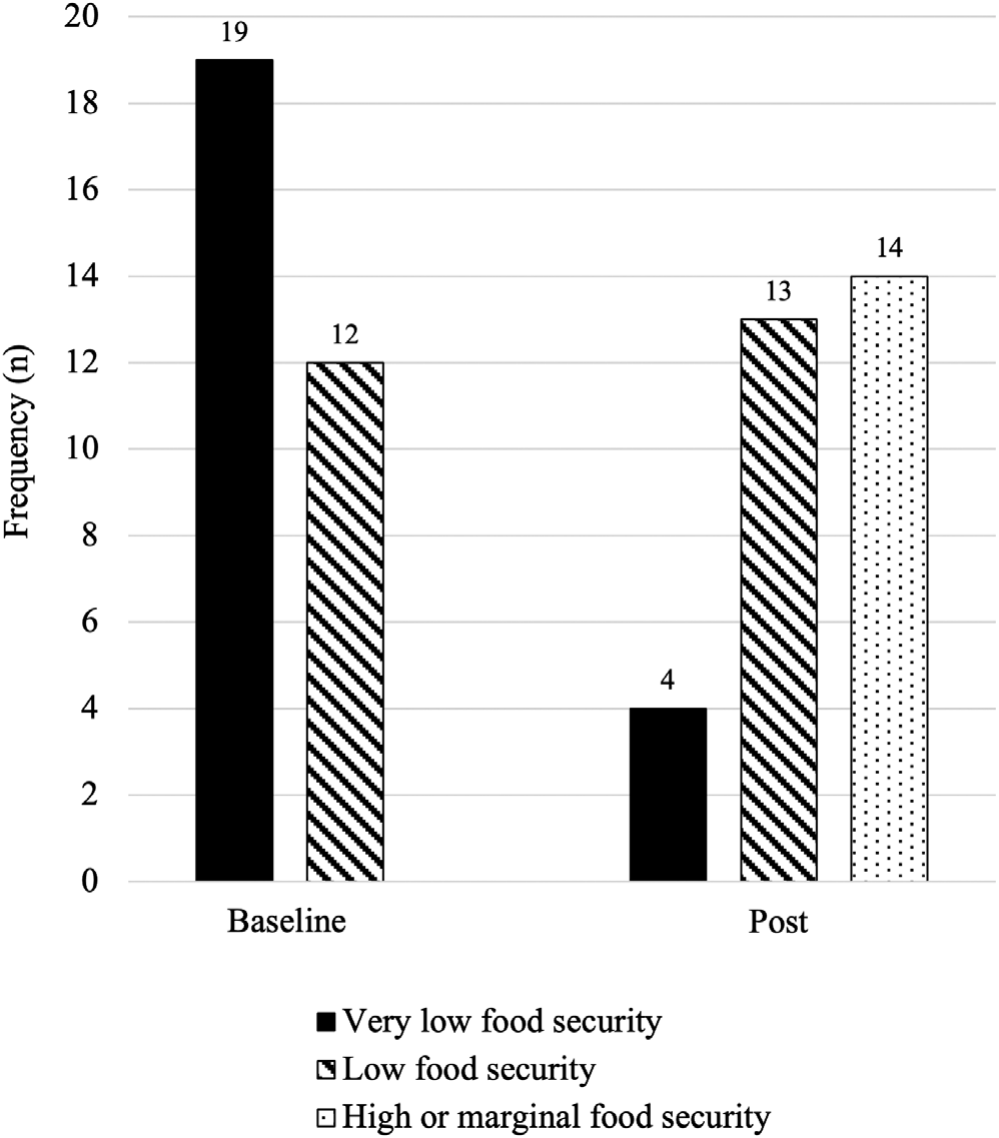
Change in level of food security status from baseline to post‐intervention.

Clinical outcome results are in Table 2. Average binge episodes decreased from baseline to post‐intervention. At post‐intervention, 71% of the sample achieved ≥ 50% reduction in binge eating, and 26% were abstinent (i.e., zero episodes in the past 28 days). Average weight decreased from baseline to post‐intervention. On average, participants lost 3.35% (SD = 3.15%) of their body weight, 52% (*n* = 16) achieved ≥ 3% reduction in weight, and 32% (*n* = 10) achieved ≥ 5% reduction in weight. Past 30‐day food security status was improved at post‐intervention (see Figure 2).

**TABLE 2:** Estimated effects of intervention on outcomes.

Measure	Baseline mean [95% CI]	Mid‐point mean [95% CI]	Post mean [95% CI]	Estimate [95% CI]	SE	*T* value	*η* ^2^ [95% CI]	Adjusted *p*
Binge eating episodes (EDE)	12.68 [10.14, 15.22]	—	4.81 [2.27, 7.34]	−5.57 [−7.26, −3.87]	0.87	−6.43	0.58 [0.38, 1]	< 0.001
Weight (pounds, self‐report)	244.97 [224, 266]	239.7 [219, 261]	236.81 [216, 258]	−5.77 [−7.54, −4.00]	0.9	−6.4	0.41 [0.25, 1]	< 0.001
Dietary restraint (EDE‐Q)	1.74 [1.18, 2.31]	2.45 [3.01, 8.59]	2.39 [1.83, 2.96]	0.46 [−0.05, 0.97]	0.26	1.78	0.07 [0, 1]	0.089
Eating concerns (EDE‐Q)	2.79 [2.26, 3.32]	2.04 [2.57, 7.74]	1.66 [1.13, 2.19]	−0.8 [−1.13, −0.46]	0.17	−4.66	0.27 [0.11, 1]	< 0.001
Shape concerns (EDE‐Q)	4.39 [3.87, 4.91]	3.69 [3.16, 4.21]	3.44 [2.91, 3.96]	−0.68 [−1.03, −0.32]	0.18	−3.74	0.2 [0.06, 1]	0.001
Weight concerns (EDE‐Q)	3.73 [3.31, 4.15]	3.48 [3.06, 3.91]	3.00 [2.57, 3.42]	−0.52 [−0.81, −0.23]	0.15	−3.48	0.17 [0.04, 1]	0.002
Clinical impairment (CIA)	27.32 [23.18, 31.5]	19.32 [15.18, 23.5]	13.9 [9.76, 18.0]	−9.49 [−12.47, −6.51]	1.52	−6.25	0.4 [0.23, 1]	< 0.001
Depressive symptoms (CESDR)	15.77 [13.43, 18.1]	13 [10.65 15.3]	11.32 [8.97, 13.7]	−3.15 [−5.02, −1.27]	0.96	−3.29	0.16 [0.03, 1]	0.002
Perceived stress (PSS)	21.71 [18.8, 24.6]	20.45 [17.6, 23.3]	19.23 [16.3, 22.1]	−1.76 [−3.87, 0.35]	1.08	−1.63	0.04 [0, 1]	0.108
Feelings of guilt (PFQ‐2)	11.9 [9.85, 14.0]	9.35 [7.31, 11.4]	8.42 [6.37, 10.5]	−2.46 [−3.86, −1.06]	0.71	−3.45	0.18 [0.04, 1]	0.002
Feelings of shame (PFQ‐2)	18.32 [15.0, 21.7]	14.81 [11.5, 18.2]	13.26 [9.9, 16.6]	−3.58 [−5.97, −1.20]	1.22	−2.94	0.13 [0.02, 1]	0.006

*Note:* Means with 95% confidence intervals (CI) for each time point are model adjusted (marginal means). Binge eating episodes were not assessed via the EDE at mid‐intervention.

Abbreviations: CESDR‐10 = Center of Epidemiologic Studies Depression Scale (clinical cut off = ≥ 16; Radloff 1977); CIA = Clinical Impairment Assessment (clinical cut‐off = 16; Bohn and Fairburn 2008); EDE = Eating Disorder Examination; EDE‐Q = Eating Disorder Examination Questionnaire (clinical cut‐off = 2.8; Mond et al. 2008); PFQ‐2 = Personal Feelings Questionnaire; PSS = Perceived Stress Scale (scores 14–26 = moderate stress; scores 27–40 = high stress).

Two participants reported compensatory behaviors at baseline. Both participants decreased their compensatory behaviors from baseline to post‐intervention. One participant decreased from 24 to 9 episodes of past 28‐day fasting and excessive exercise. The other participant decreased from 4 to 0 episodes of past 28‐day fasting.

Secondary outcomes are in Table 2. There were reductions in all EDE‐Q subscales except dietary restraint. Reductions also were observed in ED‐related clinical impairment, depressive symptoms, and feelings of shame and guilt. No significant changes were observed in perceived stress. Table S2 presents changes in comorbid mental health diagnoses and engagement in other treatment/interventions.

### 3.4 Stipend Usage

**FIGURE 3:**
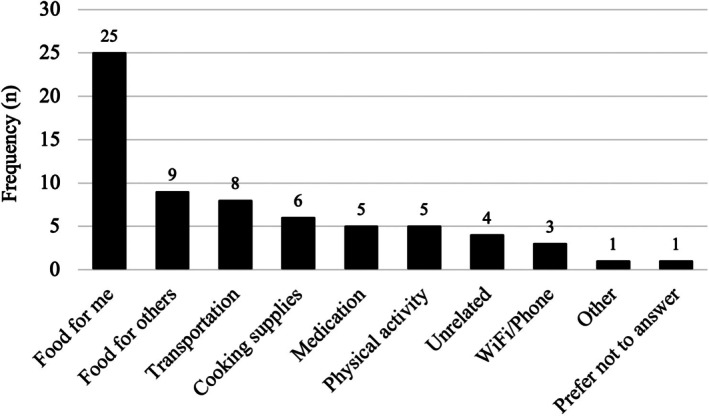
Participants' reports of how they spent their stipends. *Note:* Participants could check all that applied in their responses.

**FIGURE 4:**
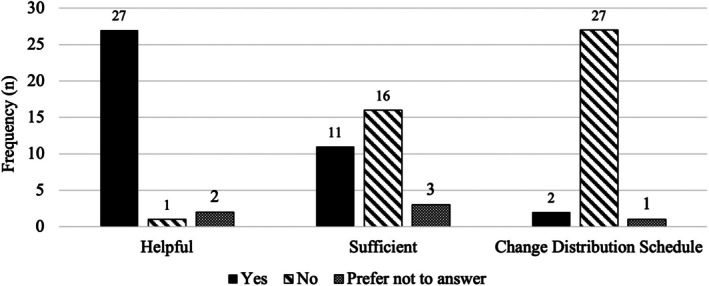
Participants' responses to stipend helpfulness, sufficiency, and distribution schedule.

Thirty participants completed the stipend questionnaire. Figure 3 shows how participants reported spending their weekly stipend. Most (83%; *n* = 25) endorsed spending on food for themselves. Fifty percent (*n* = 15) said they bought program‐related things they would not have otherwise. As Figure 4 shows, 90% (*n* = 27) said the stipend was helpful for making changes, but only 37% (*n* = 11) said it was sufficient. The average recommended increase above $10 was $45 (range = $10–$200). Yet, 53% (*n* = 16) did not want to provide a W‐9 form. Table 3 shows concerns participants endorsed about providing a W‐9 form.

**TABLE 3:** Concerns participants (*n* = 16) endorsed about having to provide a W‐9 federal payment form in order to receive their stipend via direct deposit.

Response option	*n* (%)
I worry that this could complicate how I file my taxes	7 (44%)
I have concerns about giving my personal information	7 (44%)
I worry that giving my social security number could make me lose benefits or make me ineligible for benefits	5 (31%)
I am unsure of what a W‐9 form is	4 (25%)
I am unsure of how to send you a W‐9 form	2 (13%)
I do not have a bank account	1 (6%)
I do not know my social security number, or I do not have a social security number	0 (0%)
Other reason	3 (19%)

Ninety percent (*n* = 27) said weekly distribution was their preferred deposit frequency, whereas 7% (*n* = 2) preferred monthly distribution. No participants wanted the stipend disbursed entirely at the start of the intervention or disbursed half at the start of the intervention and half at mid‐intervention. All said they would have been willing to join the program if they had to use FoodSteps‐FI to receive the stipend.

A few participants provided additional qualitative feedback, indicating the stipend was helpful for “having the extra cash when I had run out of benefits” but also that “I wish it was more” and “it helped, but healthy food is incredibly expensive.” Another participant recommended more money to “prepare menu[s] and plan meals.” Another participant said it “would be nice if participants could select their own method of payment (CashApp, PayPal, Venmo).”

## 4 Discussion

The link between food insecurity, binge eating, and weight‐related health problems like T2DM makes targeted interventions imperative, especially because barriers to treatment make help‐seeking low among people with EDs from lower socioeconomic strata (Radunz et al. 2021). This study presents results from a pilot trial of an adapted digital cognitive‐behavioral intervention for binge eating and weight‐related behaviors, FoodSteps‐FI, among people with recurrent binge eating, food insecurity, and T2DM. To our knowledge, our study is the first to test an ED intervention among this population.

### 4.1 Principal Findings

Results showed feasible recruitment, high study retention, and high intervention completion, adherence, and ratings of usability. Participants achieved improvements in binge eating, weight, and food security status, as well as in several domains of psychopathology. Although preliminary, our outcomes and engagement results are notable for a guided self‐help psychological intervention for binge eating (e.g., Traviss‐Turner et al. 2017). Further, because food insecurity does not occur in isolation, changes in clinical outcomes may have improved food security status too. Taken together, this study demonstrates the early promise of an adapted digital binge‐eating intervention for people with food insecurity and T2DM.

Although most clinical outcomes improved, dietary restraint and perceived stress did not show significant change. Non‐significant change in dietary restraint is positive for an intervention designed to improve both binge eating and weight‐related behaviors, and aligns with prior work that also has shown minimal to slight increases in EDE‐Q dietary restraint among people with binge eating and obesity who receive cognitive‐behavioral and behavioral weight loss treatments (Grilo and Pittman 2024). A lack of change in perceived stress highlights a clinical target that requires greater attention in future iterations of FoodSteps‐FI. Stress is a potential mechanism linking food insecurity and ED psychopathology (Green et al. 2025; Kosmas et al. 2023), suggesting that addressing stress within this population may reduce maladaptive behaviors.

People with EDs face a treatment gap (Fitzsimmons‐Craft et al. 2019; Kazdin et al. 2017), which is widened for those from lower socioeconomic strata (Radunz et al. 2021), making interventions warranted that improve accessibility of ED services. This study focused on a digital intervention, a delivery modality that has potential for improved accessibility and greater capacity than face‐to‐face interventions to provide real‐time support. Yet digital interventions also can pose equity challenges regarding who has access to adequate data and messaging plans that can support effective intervention engagement. Smartphone ownership, which is nearly ubiquitous among US adults (91% in 2024; Pew Research Center 2024b), does not necessarily translate to ubiquitous capacity to engage with an intervention's digital components—indeed, lower‐income households often face digital disparities such as no at‐home internet (Curtis et al. 2022; Pew Research Center 2024a). In our study, we required participants to have smartphones with sufficient data plans for calls and messaging, a potential limitation for the generalizability of our sample, and it is possible our advertisements for a digital intervention biased interest or ability to participate and thus negatively impacted trial recruitment and sample generalizability. Nevertheless, our human‐centered design process resulted in an app that requires minimal in‐app activity on a daily/weekly basis to conserve data usage and promote more frequent engagement and thus skill practice—an approach that aligns with best practices for creating equitable digital interventions (Lattie et al. 2022; Stiles‐Shields et al. 2022). Future research should continue prioritizing equity‐oriented digital interventions, including investigating the treatment needs of individuals who do not present to or are unable to engage in digital interventions to learn ways to extend relevant, accessible care to these individuals.

### 4.2 Future Directions

Our promising results indicate the need for a larger, fully powered clinical trial compared to a control condition to establish the efficacy of FoodSteps‐FI. Additionally, successful enrollment suggests devoting future attention to identifying optimal implementation settings for interventions like FoodSteps‐FI. We applied several strategies to recruit a diverse sample and continue to investigate how to reach individuals with food insecurity and binge eating for ED intervention (Graham et al. 2024; Rooper et al. 2025). Further research to answer implementation questions alongside studying intervention effectiveness can improve intervention accessibility in this population who face barriers to care.

However, despite successful enrollment, most people who completed our screener did not qualify. Although there is an increased emphasis in our field on optimizing ED interventions for previously overlooked populations (e.g., Goel et al. 2022), a consequence of establishing interventions for such populations is it can introduce more opportunities for trial exclusion. Future work could focus on pragmatic samples that enable larger inclusion criteria or on optimizing recruitment pathways to better target the right individuals.

Future research also would benefit from studying optimal stipend amounts and distribution schedules, and their feasibility in practice, including when offered in tandem with an intervention like FoodSteps‐FI, and their impact on binge eating and food security. Such research that is grounded in strategic science principles (Austin and Raffoul 2025; Austin 2016; Roberto and Brownell 2017) could have direct implications for policies (e.g., nutrition assistance benefits distribution) that impact the eating behaviors of people with food insecurity.

### 4.3 Limitations

Our sample was limited to people with T2DM and those with sufficient smartphone plans to engage in the intervention, factors that hinder sample generalizability. Further, although individuals with T2DM were included, FoodSteps‐FI did not target diabetes management beyond positive health behaviors and behavioral weight management (American Diabetes Association Professional Practice Committee 2023, 2024). We also did not assess participants' T2DM management prior to the study, nor measure diabetes‐related outcomes or changes to participants' diet and health behaviors, which would be beneficial to examine in a future, fully powered trial. Additionally, this study was a pilot trial among a small sample without a control group and no follow‐up period, requiring caution in interpreting the findings and future research to replicate our results over a longer duration. Although many of the measures had good internal consistency throughout the study, the EDEQ Weight Concerns subscale's internal consistency at baseline was low. Due to the study's eligibility criteria (e.g., a desire to lose weight), some items on this subscale may not have covaried strongly across individuals. Furthermore, the inherent instability of reliability estimates for small samples (*N* = 31) may have contributed to this result. While alpha estimates for this subscale at follow‐up timepoints were higher and stable, results nonetheless should be interpreted cautiously. Additional limitations include that we did not assess what foods participants bought with their stipend, nor other social determinants of health. Finally, while food security status showed improvement, additional research should investigate other factors that may have contributed to this improvement and whether changes are sustained post‐intervention.

## 5 Conclusion

In this pilot trial, we demonstrated that a digital intervention for binge eating, adapted to address food insecurity‐related ED behaviors, was feasible to deliver and yielded significant improvements in binge eating, weight, self‐reported rating of past 30‐day food security status, and ED‐related psychopathology among adults with food insecurity, recurrent binge eating, and T2DM. These preliminary results indicate the need for future research on the effectiveness and implementation of this and other scalable interventions for this population.

## Author Contributions

**Andrea K. Graham:** conceptualization, funding acquisition, writing – original draft, supervision, writing – review and editing, investigation, methodology, project administration.

## Funding

This work was supported by a pilot grant from the Chicago Center for Diabetes Translation Research (P30DK092949) and the Dean's Office of the Biological Sciences Division of the University of Chicago and Feinberg School of Medicine at Northwestern University, as well as by T32MH115882 from the National Institutes of Health.

## Ethics Statement

This study was approved by the Northwestern University Institutional Review Board, and all participants provided informed consent.

## Conflicts of Interest

Dr. Andrea K. Graham received grant funding from the National Institutes of Health to study digital interventions for binge eating, which included a grant in collaboration with a commercial company (R41 MH134704). The other authors declare no conflicts of interest.

## Supporting information


**Table S1:** Cronbach's alpha (α) scores for study surveys.

**Table S2:** Mental health diagnoses and additional treatment.

## Data Availability

The data that support the findings of this study are available from the corresponding author upon reasonable request.
