# Supplementary material for: Feasibility and Preliminary Efficacy of a Guided Self‐Help Digital Intervention for Adults With Food Insecurity, Recurrent Binge Eating, and Type 2 Diabetes Mellitus: A Pilot Trial
Source: Int J Eat Disord. 2026 Mar 23;59(7):1531–42. doi: 10.1002/eat.70087 (PMC13326806; doi:10.1002/eat.70087)
Supplement: Supplementary file 1 — Table S1: Cronbach's alpha (α) scores for study surveys. [file EAT-59-1531-s002.docx]

**Supplement 1.** Cronbach’s Alpha (α) Scores for Study Surveys

|  | **Study Timepoint** | | |
| --- | --- | --- | --- |
|  | Baseline  (n = 31) | Mid-Intervention  (n = 31) | Post-Intervention  (n = 30) |
| Dietary restraint (EDE-Q) | 0.84 | 0.81 | 0.82 |
| Eating concerns (EDE-Q) | 0.66 | 0.87 | 0.85 |
| Shape concerns (EDE-Q) | 0.77 | 0.91 | 0.89 |
| Weight concerns (EDE-Q) | 0.18 | 0.68 | 0.69 |
| Clinical impairment (CIA) | 0.91 | 0.94 | 0.95 |
| Depressive symptoms (CESDR) | 0.83 | 0.86 | 0.76 |
| Perceived stress (PSS) | 0.86 | 0.91 | 0.82 |
| Feelings of guilt (PFQ-2) | 0.85 | 0.85 | 0.92 |
| Feelings of shame (PFQ-2) | 0.89 | 0.89 | 0.91 |
| Food Insecurity | 0.67 | 0.83 | 0.75 |
| System Usability Scale (SUS) | -- | 0.85 | 0.81 |
