# Supplementary material for: Feasibility and Preliminary Efficacy of a Guided Self‐Help Digital Intervention for Adults With Food Insecurity, Recurrent Binge Eating, and Type 2 Diabetes Mellitus: A Pilot Trial
Source: Int J Eat Disord. 2026 Mar 23;59(7):1531–42. doi: 10.1002/eat.70087 (PMC13326806; doi:10.1002/eat.70087)
Supplement: Supplementary file 2 — Table S2: Mental health diagnoses and additional treatment. [file EAT-59-1531-s001.docx]

**Supplement 2.** Mental Health Diagnoses and Additional Treatment

|  | **Study Timepoint** | | |
| --- | --- | --- | --- |
|  | Baseline  (n = 31) | Mid-Intervention (n = 31) | Post-Intervention (n = 30) |
| **Current Diagnosis, *n* (%)** |  |  |  |
| Major Depressive Disorder/Depression | 4 (13%) | 0 (0%) | 1 (3%) |
| Anxiety Disorder | 9 (29%) | 0 (0%) | 0 (0%) |
| Bipolar Disorder/Manic Depression/Mania | 0 (0%) | 0 (0%) | 0 (0%) |
| Obsessive-compulsive disorder | 1 (3%) | 0 (0%) | 0 (0%) |
| Post Traumatic Stress Disorder | 3 (10%) | 0 (0%) | 1 (3%) |
| Dissociative disorder | 0 (0%) | 0 (0%) | 0 (0%) |
| Schizophrenia/psychotic disorder | 0 (0%) | 0 (0%) | 0 (0%) |
| Eating disorder (e.g., anorexia nervosa, bulimia nervosa, binge eating disorder) | 1 (3%) | 0 (0%) | 1 (3%) |
| Substance use disorder | 0 (0%) | 0 (0%) | 0 (0%) |
| **Current Additional Treatment, *n* (%)** |  |  |  |
| Receiving professional clinical services for management of weight or binge eating | 0 (0%) | 1 (3%) | 2 (7%) |
| Taking medication for binge eating, weight, or diabetes | 17 (55%) | 19 (61%) | 17 (57%) |
| Using apps to manage weight or binge eating, besides FoodSteps | 2 (6%) | 2 (6%) | 2 (7%) |
| Using books or workbooks (without a coach or clinician) to manage weight or binge eating | 0 (0%) | 0 (0%) | 0 (0%) |

*Note.* Diagnoses at mid-intervention and post-intervention represent those who since their last assessment, had been diagnosed with the indicated diagnoses from a mental health provider or physician.
